# Treatment patterns, resource use and costs of idiopathic pulmonary fibrosis in Spain – results of a Delphi Panel

**DOI:** 10.1186/s12890-016-0168-6

**Published:** 2016-01-12

**Authors:** Ferran Morell, Dirk Esser, Jonathan Lim, Susanne Stowasser, Alba Villacampa, Diana Nieves, Max Brosa

**Affiliations:** Vall d´Hebron Institut de Recerca (VHIR), Respiratory Department, Hospital Universitari Vall d´Hebron and CIBER in Respiratory Diseases, Passeig de la Vall d’Hebron, 119-129, 08035 Barcelona, Spain; Boehringer Ingelheim, Binger Str. 173, 55216 Ingelheim am Rhein, Germany; Oblikue Consulting S.L., Avenida Diagonal 514, 3°-3a, 08006 Barcelona, Spain

**Keywords:** Costs, Delphi technique, Idiopathic pulmonary fibrosis, Spain

## Abstract

**Background:**

Idiopathic pulmonary fibrosis (IPF) is a form of chronic fibrosing interstitial pneumonia characterized by progressive worsening of dyspnea and lung function, with a poor prognosis. The objective of this study was to determine treatment patterns, resource use and costs of managing Spanish patients with IPF.

**Methods:**

A three-round Delphi consensus panel of 15 clinical experts was held between December 2012 and June 2013 using questionnaires to describe the management of patients with IPF. A cost analysis based on Delphi panel estimates was made from the Spanish National Health System (NHS) perspective, including the direct costs of IPF diagnosis and management. Unit costs were applied to Delphi panel estimates of health resource use. Univariate sensitivity analyses were made to evaluate uncertainties in parameters.

**Results:**

The Delphi panel estimated that 20, 60 and 20 % of IPF patients presented with stable disease, slow and rapid disease progression, respectively. The estimated annual cost per patient with stable disease, slow and rapid disease progression was €11,484, €20,978 and €57,759, respectively. This corresponds to a weighted average annual cost of €26,435 with itemized costs of €1,184 (4.5), €7,147 (27.0), €5,950 (22.5), €11,666 (44.1) and €488 (1.9 %) for the diagnosis of IPF, treatment, monitoring, management of acute exacerbations and end-of-life care, respectively. The parameter that varied the annual cost per patient the most was resource use associated with acute exacerbations.

**Conclusions:**

The management of patients with IPF in Spain, especially patients with rapid disease progression, has a high economic impact on the NHS.

**Electronic supplementary material:**

The online version of this article (doi:10.1186/s12890-016-0168-6) contains supplementary material, which is available to authorized users.

## Background

Idiopathic pulmonary fibrosis (IPF) is a specific form of chronic, progressive fibrosing interstitial pneumonia associated with the histopathological and/or radiological pattern of usual interstitial pneumonia (UIP) [1]. A diagnosis of IPF requires the exclusion of other forms of interstitial lung disease (ILD) such as those associated with environmental exposure, medication or systemic disease [1]. IPF usually affects adults aged >50 years; the prevalence is higher in males and most patients have a history of smoking [1–3].

IPF is characterised by progressive worsening of dyspnoea and lung function, with a poor prognosis, but the natural history of the disease is variable and unpredictable in the individual patient [1]. In most patients, IPF worsens slowly but steadily (“slow progression”), in some it remains stable (“stable”) and in some it worsens rapidly (“rapid progression”) [1]. In addition, some patients experience acute exacerbations, defined as episodes of acute respiratory worsening of unknown cause [4].

In the past, IPF was considered to have an inflammatory origin and, consequently, anti-inflammatory and immunosuppressive drugs have been used [5, 6]. However, the central event currently considered to be involved in the development of IPF is repeated alveolar epithelial cell injury resulting in an impaired repair process with development of fibrosis with inflammation now considered less important [7]. This scenario has encouraged the development of new drugs with antifibrotic properties [8].

The overall economic burden associated with IPF in Spain remains unknown. Studies analysing the cost of illness could help to define the magnitude of economic burden associated with IPF and its impact on Spanish society. The objective of this study, which used a Delphi panel, was to determine treatment patterns, resource use and the associated costs of managing patients with IPF in the Spanish healthcare system.

## Methods

### Delphi panel

A three-round Delphi panel of clinical experts was held between December 2012 and June 2013 using postal questionnaires to describe the treatment patterns and resource use associated with IPF. The first questionnaire included questions on the clinical management of patients with IPF in Spain and open questions that investigated several aspects of the disease. The second questionnaire sought to study specific issues in greater detail and to resolve any doubts about questions asked in the first questionnaire. The third questionnaire was carried out to reach a consensus among the experts. An aggregate of the three questionnaires is available on the journal website (Additional file 1). In each round, the participants were asked the questions individually. All participants remained anonymous until the end of the process.

Fifteen pulmonologists who were experts on ILD were recruited from the Spanish respiratory community to ensure that the findings of the Delphi panel were credible and reflective of clinical practice in Spain. Pulmonologists were recruited from different Spanish Autonomous Communities to guarantee that regional differences were captured (Andalusia, Asturias, Castile-La Mancha, Castile-Leon, Catalonia, Community of Madrid, Valencian Community, Galicia) (see Acknowledgements). The study sponsor did not influence selection of the panelists and was unaware of their identity until study completion. Study panelists were unaware of the identity of the study sponsor. The study did not require approval from an ethics committee due to its design [9].

### Cost analysis

A cost analysis was made from the Spanish National Health System (NHS) perspective, based on data obtained through a systematic literature review and the Delphi panel estimates of the number of patients with IPF in Spain and their resource use during diagnosis, treatment (pharmacological and non-pharmacological) and management (medical visits, tests, other resources). All costs were estimated per patient per year, and were specified with respect to the different types of disease course. To calculate an overall annual cost per patient, the estimated cost associated with each type of disease course was weighted by its proportion of all patients. Costs were expressed in 2013 euro.

### Unit costs

Unit costs were obtained from Spanish databases (see Additional file 2: Table S1, Table 1). Pharmacological costs were obtained from the Spanish database of the Consejo General de Colegios Oficiales de Farmacéuticos, and all costs included were ex-factory price [10]. Unit costs related to medical visits, tests, hospital admissions, etc. were obtained from the eSalud Spanish health costs database [11]. Diagnostic-related group costs were obtained from the Spanish Ministry of Health database [12].

**Table 1:** Cost of management of adverse events associated with the treatment of IPF

	Non-hospitalised grade 3 adverse events^a^	Grade 3 and 4 adverse events in hospitalised patients
Osteoporosis	€89.43	€3,495.47
Hyperglycaemia	€69.93	€3,898.39
Cushing syndrome	€74.66	€4,468.42
Compression fractures	€74.66	€3,495.47
Diabetes	€84.74	€3,898.39
Digestive intolerance	€68.14	€2,452.50
Opportunistic infections	€74.66	€7,085.52
Hepatotoxicity	€74.66	€2,739.44
Nausea	€68.14	€ 3,082.73
Digestive intolerance	€74.66	^b^
Nasal dryness	€27.95	€1,678.20
Asthenia	^b^	€3,082.73

Source: eSalud [11]

^a^Costs were estimated taking into account medical visits and tests undergone by patients for their management

^b^These adverse events were reported by <5 % of patients therefore these costs were not included

### Types of disease course in IPF

The expert panel estimated the proportion of patients with IPF with stable disease, slow and rapid disease progression, according to the types of disease courses described in the ATS/ERS/JRS/ALAT Statement [1]. Treatment and monitoring costs were estimated according to the type of disease course. The costs associated with an acute exacerbation were assumed to be the same for all types of patients. The annual cost for each type of disease course was calculated by weighting the cost according to the number of exacerbations per each type of patient per year. A similar approach was used to estimate costs associated with end-of-life care. The cost per year associated with end-of-life care was weighted by the survival rates observed per type of disease course.

### Epidemiological approach

The cost of illness analysis was performed using a prevalence-based approach, as this method allows disease-attributable costs that occur concurrently with prevalent cases over a specified time period (1 year in this case) to be measured [13]. To calculate the current number of patients with IPF in Spain, the median prevalence obtained through the Delphi panel was multiplied by the Spanish population obtained from the Spanish National Statistics Institute [14].

### Diagnostic costs

To calculate the number of patients diagnosed with IPF, the diagnostic rate was assumed to be 85 %, based on a series of patients with clinical characteristics of ILD [15], This rate was applied to the number of Spanish patients estimated by the Delphi panel. To estimate the total cost associated with the diagnosis of IPF, the median number of medical visits or tests carried out to reach the diagnosis was multiplied by their mean unit costs. To determine the cost of diagnosis per year, the estimated total cost associated with the diagnosis of IPF was multiplied by the ratio of incidence over prevalence.

### Treatment of patients with IPF by disease course

The cost associated with pharmacological therapies included drugs and the cost of drug-related adverse events. The mean cost per unit and the daily median dose administered were multiplied by the median treatment duration. Only drug-related adverse events affecting >5 % of patients were included and costs associated with Common Terminology Criteria for Adverse Events grade 1–2 (mild and moderate) adverse events were excluded [16]. The cost associated with grade 3 (severe) adverse events included the cost of patients hospitalised due to adverse events and resource use by non-hospitalised patients (Table 1). It was assumed that all patients suffering grade 4 (potentially life threatening) adverse events were hospitalised. To calculate the costs associated with non-pharmacological therapies, the percentages of patients undergoing lung transplantation or pulmonary rehabilitation were multiplied by their unit costs (see Additional file 2: Table S1).

### Monitoring by clinical disease course per year

To estimate the cost associated with IPF monitoring, the mean unit costs of medical visits, tests and hospital admissions (see Additional file 2: Table S1) were multiplied by the median number of resources used per type of patient and year according to estimates from the Delphi panel.

### Management of patients during acute exacerbations

The total number of acute exacerbations suffered per type of patient/year was calculated to estimate the costs associated with their management per year according to the estimates of the Delphi panel. Diagnosis, treatment, healthcare resource use and follow-up costs were included. To calculate the cost associated with diagnosis, unit costs related to tests carried out to differentiate acute exacerbations from other causes of acute respiratory failure or clinical deterioration were multiplied by the percentage of patients undergoing each test as estimated by the Delphi panel. Treatment costs associated with acute exacerbations were included. To calculate the costs associated with each therapeutic regimen, the mean cost per unit and the daily median dose were multiplied by the duration of treatment. The associated costs of healthcare resource use and follow-up during an acute exacerbation were estimated, and the unit costs of medical visits and tests were multiplied by the percentage of patients receiving them. To estimate the overall cost per year associated with acute exacerbations for each type of disease course, the total cost of an acute exacerbation was multiplied by the number of acute exacerbations suffered per year by each type of patient.

### End-of-life care

Treatment costs were calculated by multiplying mean unit costs, median doses administered and the duration of each treatment by the percentage of patients receiving each treatment according to Delphi panel estimates. To calculate the costs associated with outpatient visits, the median number of visits per patient was multiplied by their unit cost. To distribute end-of-life care costs annually, the annual mortality rate per type of disease course was multiplied by the total end-of-life cost, in order to weight this cost by the proportion of patients who receive end-of-life care per year. The annual mortality rate was calculated using a DEALE approximation (Declining Exponential Approximation to Life Expectancy) [17].

### Sensitivity analysis

Successive univariate sensitivity analyses were performed on key values in the cost analysis to ascertain the circumstances under which uncertainty or lack of agreement about any estimate may significantly impact the results. Specific parameters were varied one at a time across a plausible range, while the remaining values were held at baseline values. The parameters varied were the prevalence and incidence estimates made by the Delphi panel (±25 %), resource use associated with the management of IPF derived from the Delphi panel (minimum and maximum values), and unit costs obtained from Spanish databases (minimum and maximum values).

## Results

### Delphi panel

The first round Delphi Panel was completed by 15 pulmonologists and the second and third rounds by 14. The degree of consensus in the third questionnaire was high, with 77–100 % agreement per question.

Epidemiological estimates by the Delphi panel suggested that the prevalence of IPF in Spain is 12 cases per 100,000 people/year and the incidence is 3 cases per 100,000 people/year. Based on a Spanish population of 46,039,979, it was calculated that there are 5,525 people with IPF in Spain.

Considering a diagnostic rate of 85 % [15], the number of diagnosed patients with IPF in Spain was estimated to be 4,696. The Delphi panel estimated that two primary care visits, three pulmonary medicine department visits, multiple laboratory tests and other tests such as chest x-ray, high resolution computed tomography (HRCT), bronchoscopy, bronchoalveolar lavage, transbronchial biopsy, surgical lung biopsy, blood gases and respiratory function tests were carried out to reach a diagnosis of IPF.

It was estimated that 20, 60 and 20 % of IPF patients presented with stable disease, slow and rapid disease progression, respectively, and that mean survival after diagnosis was 66, 42 and 15 months, respectively.

The Delphi panel estimated that most patients with IPF are administered N-acetylcysteine (Table 2). All patients with rapid disease progression also receive long-term oxygen therapy and half are treated with prednisone (Table 2). N-acetylcysteine does not induce grade 3–4 adverse events (Table 3). The main grade 3–4 adverse events associated with prednisone are osteoporosis, myopathy, hyperglycemia, Cushing syndrome and diabetes (Table 3). The main grade 1–4 adverse event associated with long-term oxygen therapy is nasal dryness (Table 3). Some patients are also treated with non-pharmacological therapies such as pulmonary rehabilitation (10 % of patients) and single-lung transplant (5 and 8 % of patients with slow and rapid disease progression, respectively) (Table 2).

**Table 2:** Treatment of patients with IPF by clinical disease course

	Stable disease	Slow disease progression	Rapid disease progression
Pharmacological			
N-acetylcysteine	80 %	100 %	100 %
Anticoagulants	0 %	0 %	4.3 %
Prednisone	0 %	0 %	50 %
Long-term oxygen therapy	25 %	30 %	100 %
Omeprazole or pantoprazole	7 %	7 %	7 %
Pirfenidone (compassionate use /importation)	0 %	5 %	0 %
Non-pharmacological			
Single-lung transplant	0 %	5 %	8 %
Pulmonary rehabilitation	10 %	10 %	10 %

**Table 3:** Adverse events associated with IPF treatments

	% of patients		
	Grade 1–2	Grade 3	Grade 4
N-Acetylcysteine			
Epigastric pain	5 %	0 %	0 %
Digestive intolerance	8 %	0 %	0 %
Dyspepsia	5 %	0 %	0 %
Reflux	1 %	0 %	0 %
Nausea	1 %	0 %	0 %
Anticoagulants			
Haematoma	10 %	0 %	0 %
Systemic corticosteroids			
Osteoporosis	30 %	18 %	5 %
Opportunistic infections	10 %	5 %	1 %
Oedema	10 %	3 %	0 %
Myopathy	15 %	5 %	5 %
Hyperglycaemia	30 %	18 %	5 %
Cushing syndrome	20 %	10 %	5 %
Compression fractures	10 %	10 %	3 %
Diabetes	15 %	10 %	5 %
Hypertension	10 %	5 %	0 %
Cataracts	8 %	5 %	1 %
Digestive intolerance	10 %	7 %	3 %
Pirfenidone			
Photosensitivity	20 %	0 %	0 %
Epigastric pain	15 %	3 %	0 %
Skin reactions	15 %	0 %	0 %
Nausea	8 %	10 %	0 %
Asthenia	10 %	3 %	0 %
Digestive intolerance	10 %	8 %	0 %
Long-term oxygen therapy			
Nasal dryness	40 %	10 %	3 %
Epistaxis	10 %	0 %	0 %
Dry mouth	8 %	3 %	0 %

Table 4 shows that patients with rapid disease progression used the most healthcare resources.

**Table 4:** Median healthcare resource use for managing acute exacerbations and follow-up of patients with IPF

	Acute exacerbation and follow-up	Management and follow-up of patients with IPF (over a 3-month period)		
		Stable disease	Slow disease progression	Rapid disease progression
Outpatient visits				
General practitioner home visits	0.2	0.0	0.0	0.0
Pulmonary specialist	2.0	1.0	1.0	2.0
Nurse (or other healthcare professional)	0.6	0.0	0.0	0.0
Elective ambulance	0.3	0.0	0.0	0.0
Emergency				
Emergency room visits	1.0	0.0	0.0	1.0
Emergency ambulance	0.8	0.0	0.0	0.0
Hospital admissions				
Pulmonary department (days)	11.3	0.0	0.0	7.5
Intensive care unit (days)	2.5	0.0	0.0	0.8
Laboratory tests				
Complete blood count	3.0	1.0	1.0	1.0
Sedimentation rate	2.0	0.0	1.0	1.0
Liver profile	2.0	0.0	1.0	1.0
Creatine phosphokinase	1.5	0.0	0.0	1.0
Urinalysis	0.2	0.0	0.0	0.0
Microbiology	0.1	0.0	0.0	0.0
Respiratory function tests				
Spirometry	1.0	1.0	1.0	1.0
Body plethysmography	0.1	0.0	1.0	1.0
Diffusing capacity of carbon monoxide	1.0	0.0	1.0	1.0
6-min walk test	0.3	0.0	1.0	1.0
Other tests				
Chest X-ray	3.0	0.0	0.5	1.0
High-resolution computed tomography	1.0	0.0	0.0	1.0
Blood gases	3.0	0.0	0.3	1.5
Computed tomography pulmonary angiogram	0.3	0.0	0.0	0.0
Bronchoscopy	0.3	0.0	0.0	0.0
Sputum assessment	0.8	0.0	0.0	0.0
Bronchoalveolar lavage	0.2	0.0	0.0	0.0

The Delphi panel estimated that the number of exacerbations per patient/year was 0.76 in patients with stable disease, 0.82 in patients with slow disease progression and 1.8 in patients with rapid progression. The panel estimated that 51 % of patients with IPF with an acute exacerbation die from this event. Of these, 69 die in the hospital and 31 % during the 6 months after hospital discharge. Tests performed in more than 50 % of patients with IPF to differentiate an acute exacerbation from other causes of acute respiratory failure or clinical deterioration included computed tomography, echocardiography and laboratory tests. Table 4 shows that, during an acute exacerbation and follow-up, patients use considerable healthcare resources, including medical visits, hospitalisations and multiple tests. Treatments administered to treat acute exacerbations were corticosteroids (100 of patients), antibiotics (93), and anticoagulant drugs (79) and non-invasive (79) and invasive (71 %) mechanical ventilation.

The Delphi panel estimated that, during end-of-life care, most physicians initiate palliative care when patients with IPF develop uncontrollable dyspnoea. Annual mortality rates were 18.2, 28.6 and 80 % in patients with stable disease, slow and rapid disease progression, respectively. The median duration of palliative treatment was 4.5 months. The main active ingredients administered as palliative treatment were paracetamol (50 of patients), codeine (40), morphine (40) and corticosteroids (20 %).

### Costs

The total cost of the diagnosis of IPF was €4,736 per patient. When this cost was distributed by the proportion of new patients diagnosed every year (by multiplying with the ratio of incidence over prevalence), there was an annual cost of €1,184 per patient (Table 5). The main cost drivers were surgical lung biopsy, bronchoscopy with bronchoalveolar lavage and transbronchial biopsy and pulmonary medicine department visits.

**Table 5:** Total cost per patient per year according to disease course

	Stable disease	Slow disease progression	Rapid disease progression	Total cost weighted by type of disease distribution
Diagnosis	€1,184.07	€1,184.07	€1,184.07	€1,184.07
Treatment	€722.26	€8,069.33	€10,802.52	€7,146.55
Monitoring	€453.94	€1,698.91	€24,199.00	€5,949.93
Acute exacerbations	€8,882.22	€9,646.21	€20,511.50	€11,666.47
End-of-life care	€241.28	€379.15	€1,061.62	€488.07
TOTAL	€11,483.76	€20,977.67	€57,758.70	€26,435.10

The mean associated cost of treatment per patient/year was €722 for patients with stable disease, €8,069 for patients with slow disease progression and €10,803 for patients with rapid disease progression (Table 5). The high cost in patients with slow disease progression and rapid disease progression was mainly attributable to the cost of lung transplantation. When total costs per patient were weighted by the proportion of patients with each type of disease course, an overall cost of €7,147 was obtained per patient/year (Table 5).

The annual mean costs associated with IPF monitoring were €454, €1,699 and €24,199 per patient with stable disease, slow and rapid disease progression, respectively (Table 5). The most costly resources were those associated with pulmonary specialist visits and spirometry tests for patients with stable disease, diffusing capacity of carbon monoxide and 6-min walk tests for patients with slow disease progression, and hospital admissions for patients with rapid disease progression. The cost weighted by types of disease course showed an overall cost of €5,950 per IPF patient/year (Table 5).

The cost of a diagnosis of an acute exacerbation was €339, the overall treatment cost was €305 (corticosteroids, antibiotics, and anticoagulants and non-invasive ventilation) and the cost of healthcare resource use and follow-up during an acute exacerbation was €11,074 per patient. Therefore, the total cost of an acute exacerbation was €11,718, with healthcare resource use and follow-up representing 95 % of the total cost. The overall cost associated with acute exacerbations per year was €8,882 per patient with stable disease, €9,646 per patient with slow disease progression and €20,511 per patient with rapid disease progression (Table 5). Weighting these costs per type of disease course showed a global cost of €11,666 per patient per year (Table 5).

The mean total cost of end-of-life care treatment was €463 per patient, regardless of the type of disease course. The cost of outpatient visits during end-of-life care was €864 per patient. Therefore, considering both treatment and medical visits, the total cost of end-of-life care was €1,327 per patient. Considering the annual mortality rate for each type of disease course, the mean associated cost of end-of-life care per year was €241 for patients with stable disease, €379 for patients with slow disease progression and €1,062 for patients with rapid disease progression (Table 5). The cost weighted by types of disease course showed an overall cost of €488 per patient per year (Table 5).

**Fig. 1:**
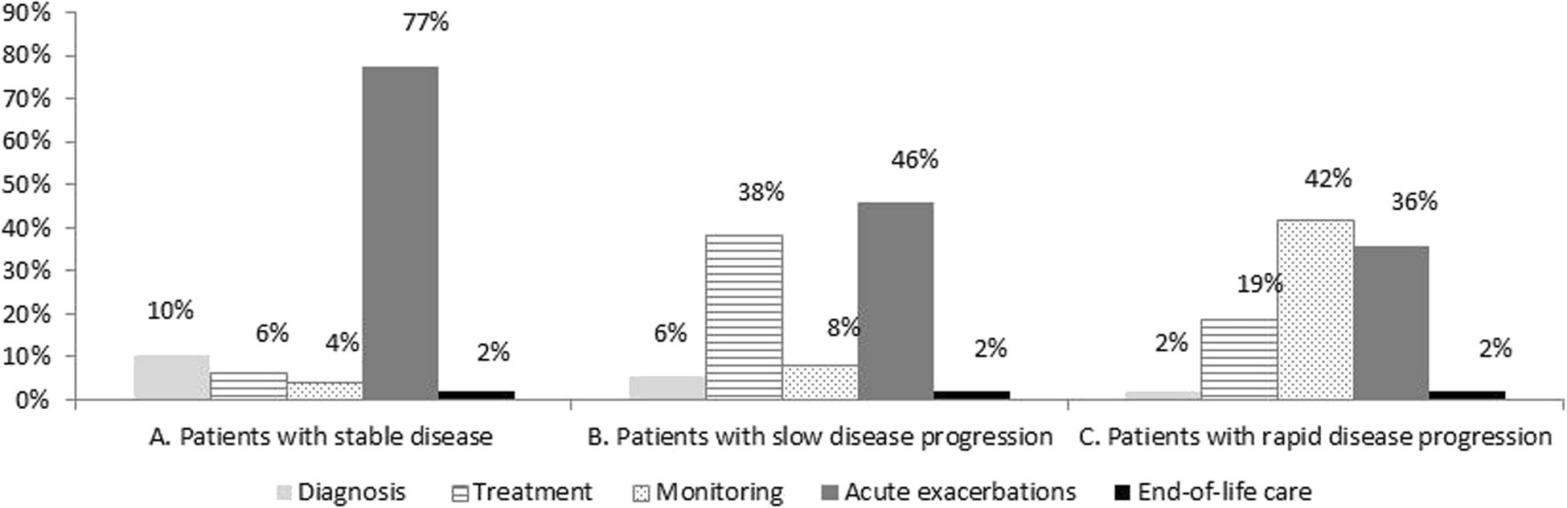
Costs shown as a percentage of the overall cost by type of disease course

Taking into consideration all the factors studied, a total cost of €11,484, €20,978 and €57,759 were obtained per year per patient with stable disease, slow disease progression and rapid disease progression, respectively (Table 5). Therefore, the cost in patients with rapid disease progression was about 5 times higher than that for patients with stable disease and 3 times higher than that for patients with slow disease progression. Weighting these costs by the proportion of patients representing each type of clinical disease course resulted in a mean cost of €26,435 per patient per year (Table 5; Fig. 1).

### Sensitivity analysis

Univariate sensitivity analyses of the prevalence, incidence, resource use and cost data were made to calculate changes in total costs when these parameters were varied. The main parameters that the total cost was most sensitive to were resource use during the clinical management of exacerbations, treatment and monitoring. The costs associated with treatment (pharmacological and the cost of treating adverse events) also changed the results considerably (Table 6).

**Table 6:** Univariate sensitivity analysis

	Mean cost per patient per year			
Base case	€26,435			
Parameter	Minimum	Maximum	Minimum.	Maximum.
			Difference from base case (%)	Difference from base case (%)
Prevalence	€26,830	€26,198	1 %	−1 %
Incidence	€26,139	€26,731	−1 %	1 %
Resource use during disease diagnosis	€25,349	€27,745	−4 %	5 %
Resource use during treatment by disease course	€19,408	€83,145	−27 %	215 %
Resource use during monitoring by disease course	€21,882	€51,531	−17 %	95 %
Resource use during clinical management of exacerbations	€16,882	€93,113	−36 %	252 %
Resource use during end-of-life care	€25,975	€31,351	−2 %	19 %
Cost of medical visits	€24,772	€28,574	−6 %	8 %
Cost of hospitalizations	€24,926	€28,491	−6 %	8 %
Cost of treatment	€24,492	€35,522	−7 %	34 %
Cost of tests	€24,127	€28,938	−9 %	9 %

## Discussion

This study aimed to assess treatment patterns, resource use and associated costs in Spanish patients with IPF. The results are of interest due to the lack of studies analysing the burden associated with IPF and may help to indicate medical needs in the management of patients with IPF and guide research and investment in health resources. Our study is the first detailed analysis of healthcare costs associated with the overall management of patients with IPF in Spain, from diagnosis until end-of-life care.

Our results show that the total annual costs per patient with stable disease, slow and rapid disease progression were €11,484, €20,978 and €57,759, respectively. Weighting these costs by type of disease course showed a mean cost of €26,435 per IPF patient per year. These results are comparable to those of a retrospective study of US claims databases that included 9,286 patients and found that the total direct cost of IPF was $26,378 per patient per year [18]. However, there were methodological differences between this and our study. In the US study, two cohorts (patients with IPF and matched controls) were retrospectively identified from US claims databases in order to analyse the prevalence and incidence of pre-selected comorbidities and to collect data on healthcare resource use (hospital, outpatient, drugs) in each cohort; costs were not estimated according to type of disease course. As reflected by our results, the clinical course has a considerable impact on disease management and costs of treatment. We did not analyse the incidence and prevalence of comorbidities because we considered that they are not a direct or inevitable consequence of IPF.

In Spain, some studies have evaluated the cost associated with the management of chronic lung diseases such as chronic obstructive pulmonary disease (COPD). The mean annual cost per patient with COPD (including hospital care, ambulatory care, oxygen therapy, usual drug therapy and drug therapy for exacerbations) has been estimated at around €2,000 [19–21], suggesting that the cost associated with the management of patients with IPF (€26,435 in our study) is considerably higher than other chronic lung diseases.

The sensitivity analysis showed that the parameters with the greatest impact on the results were those related to healthcare resource use during the clinical management of exacerbations, treatment and monitoring. The impact of these factors was due to variations in the estimates of resource use by the clinical experts (rather than estimated unit costs). This suggests differences in clinical practice between experts and variations in access to, or use of, healthcare resources between health centers. In addition, variations between published recommendations and clinical practice were found (for example, in the use of diagnostic tools such as bronchoalveolar lavage).

Our results may reflect true variability of the management of patients with IPF in Spanish clinical practice, likely due in part to the lack of effective pharmacological treatments. It remains to be seen how recently reported findings in IPF [22–24] and the availability of new therapeutic options will change treatment patterns in IPF. Nintedanib was not available at the time that this Delphi panel was carried out while pirfenidone had not been reimbursed and was only available through compassionate use; consideration of these treatments would probably increase the estimated economic burden associated with IPF.

Lung transplantation is the main determinant of treatment costs in patients with IPF. It might seem that the number of patients receiving lung transplantation in our study is low (5 and 8 % of patients with slow and rapid disease progression, respectively), but considering that 262 lung transplants were performed in Spain in 2014 [25] and that approximately one third of them are in patients with IPF [26], it can be extrapolated that approximately 1.3 % of patients with IPF receive a transplantation. It is also important to note that not all patients with IPF are candidates for lung transplantation [27].

The incidence and prevalence rates in our study (3 cases per 100,000 people/year and 12 cases per 100,000 people/year, respectively) are within the values in a recent review that estimated an annual incidence of IPF between 0.22 and 7.4 per 100,000 population and a prevalence between 1.25 and 23.4 cases per 100,000 population in Europe [28]. The true incidence and prevalence of IPF are difficult to estimate due to differences in coding, under- and misdiagnosis, e.g. a recent Spanish prospective observational study suggested that almost half of patients diagnosed with IPF may subsequently be diagnosed with chronic hypersensitivity pneumonitis [29].

One limitation of our study is that it was based on estimates from a Delphi panel and not on prospective or retrospective data. The Delphi panel consisted of 15 physicians who, although selected because of their ILD expertise, may not be fully representative of Spanish clinical practice. Nevertheless, it should be taken into account that experts were chosen from different regions in Spain, and since IPF is an orphan disease, the number of Spanish experts is limited. Indirect costs (e.g. work productivity) could not be determined because experts stated they lacked the information required to answer questions related to the impact of IPF on patients, relatives and caregivers. Studies assessing indirect costs would help to show the impact of IPF from the societal perspective. The study results were calculated on the basis of classifying patients as having stable disease, slow disease progression or rapid disease progression. Although these groups correspond to different possible natural histories of IPF, there is no precise definition of these terms, which may be a limitation of our study. The Delphi panel estimates did not distinguish between the outpatient and inpatient costs associated with exacerbations, treatment, monitoring and end-of-life care. This might have led to double counting and an overestimate of the costs (the cost of hospital admissions, for example, already include the cost of tests, treatments and visits). In contrast, the calculation of the cost of drug-related adverse events excluded grade 1–2 adverse effects and grade 3–4 adverse effects affecting ≤5 % of patients. Therefore, there might have been an underestimation of the cost of adverse events. Finally, the number of exacerbations per year could have been overestimated because the questionnaires of the Delphi panel did not include a specific definition for an acute exacerbation of IPF and physicians may have included worsenings of IPF with an identifiable cause. This could explain why our results are higher than other data on acute exacerbations of IPF [30]. Despite these limitations, the similarities between our results and those of other studies on the impact of IPF [18] suggest that our study may provide a reasonable estimate of the cost of IPF in Spain.

## Conclusions

The results of this study suggest that the management of patients with IPF in Spain, and especially patients with rapid disease progression, has a high economic impact on the NHS. The cost associated with acute exacerbations represents nearly half of the total cost of managing patients with IPF and therefore, the availability of new treatments that reduce the risk of acute exacerbations may reduce the economic impact of IPF. This study further supports the need for treatments modifying the course of IPF.

## Additional files

Additional file 1:
**Questionnaire aggregating the three rounds of the Delphi panel.** (DOCX 133 kb) Additional file 2: Table S1. Unit costs used in the study. (DOCX 23 kb)

## Abbreviations

COPD: Chronic obstructive pulmonary disorder
DEALE: Declining Exponential Approximation to Life Expectancy
HRCT: High resolution computed tomography
ILD: Interstitial lung disease
IPF: Idiopathic pulmonary fibrosis
NHS: National Health System
UIP: Usual interstitial pneumonia
