# Supplementary material for: Treatment patterns, resource use and costs of idiopathic pulmonary fibrosis in Spain – results of a Delphi Panel
Source: BMC Pulm Med. 2016 Jan 12;16:7. doi: 10.1186/s12890-016-0168-6 (PMC4710031; doi:10.1186/s12890-016-0168-6)
Supplement: Additional file 1: — Questionnaire aggregating the three rounds of the Delphi panel. (DOCX 133 kb) [file 12890_2016_168_MOESM1_ESM.docx]

**Additional file 1 to accompany research article “Treatment patterns, resource use and costs of idiopathic pulmonary fibrosis in Spain – results of a Delphi Panel” by Ferran Morell et al**

**QUESTIONNAIRE AGGREGATING THE 3 ROUNDS**

**Epidemiology**

**Prevalence^^[[1]](#footnote-1)^^**

**1.** Although there are no Spanish studies that analyze the prevalence of IPF in Spain, most of the articles reviewed suggest a prevalence of IPF in the general population of 13 cases per 100,000 women and 20 cases per 100,000 men (Consenso para el diagnóstico de las neumonías intersticiales idiopáticas, 2010; Ancochea et al., 2004; Vaquero, 2008; Xaubet, 2003; Xaubet et al., 2003).

Please indicate whether you agree with this data.

| YES |  |
| --- | --- |
| NO |  |

If not, please explain why you do not agree with the proposed prevalence estimates given in the national literature?

|  |
| --- |

Comments (add here any additional information you might have)

|  |
| --- |

**2.** In your experience, could you indicate how many IPF patients do you see each year?

|  |
| --- |

Comments (add here any additional information you might have)

|  |
| --- |

**Incidence^[[2]](#footnote-2)^**

**3.** Currently, there are no Spanish studies that report the incidence of IPF in Spain. The estimated incidence may range between 4.6 and 7.4 cases per 100,000 persons (Xaubet and Ancochea, 2012).

Please indicate whether you agree with this data.

| YES |  |
| --- | --- |
| No |  |

If not, please indicate why you do not agree on the proposed incidence range given in the literature and specify, according to your experience, the annual incidence range of IPF in the general population in Spain

|  |
| --- |

Comments (add here any additional information you might have)

|  |
| --- |

**4.** Could you indicate, according to your experience, how many new IPF patients do you see per year?

|  |
| --- |

Comments (add here any additional information you might have)

|  |
| --- |

**5.** In order to extrapolate epidemiological data to the Spanish population, could you indicate the reference population area of your hospital (total population assigned to your hospital)?

|  |
| --- |

Comments (add here any additional information you might have)

|  |
| --- |

**Diagnosis**

**6.** Based on your experience and the recollection of your patients, could you estimate the average time between first onset of symptoms and diagnosis of IPF (i.e. the delay in diagnosis)?

|  |
| --- |

Comments (add here any additional information you might have)

|  |
| --- |

**Resource use in the diagnosis of IPF patients**

**7.** According to usual clinical practice, could you indicate the mean number of tests and visits undertaken in order to get to a diagnosis of IPF? (Mora and Romero, 2012; Marcos PJ et al., 2012).

| Visits / Tests | Mean number of tests or visits |
| --- | --- |
| Visits made to reach a definitive diagnosis of IPF |  |
| Primary care |  |
| Pulmonary medicine department |  |
| Laboratory Tests |  |
| Complete blood count |  |
| Sedimentation rate |  |
| Hepatic profile |  |
| Creatine phosphokinase (CPK) |  |
| Angiotensin-converting enzyme (ACE) |  |
| Rheumatoid factor |  |
| Antinuclear antibodies |  |
| Urinalysis |  |
| Other tests |  |
| Chest X-ray |  |
| High resolution computed tomography (HRCT) |  |
| Computed tomography pulmonary angiogram |  |
| Bronchoscopy^[[3]](#footnote-3)^ |  |
| Sputum assessment |  |
| Bronchoalveolar lavage |  |
| Transbronchial Biopsy |  |
| Surgical lung biopsy |  |
| Ventilation/perfusion scan |  |
| Blood gases |  |
| Respiratory function tests: |  |
| Spirometry |  |
| Bodyplethysmography |  |
| Diffusing capacity of carbon monoxide |  |
| 6 minute walk test |  |
| Other (e.g. ergospirometry, cycle-ergometry,…)  ……………………… |  |
| Other (e.g. ergospirometry, cycle-ergometry, …)  ……………………… |  |

Comments (add here any additional information you might have)

|  |
| --- |

**Natural history of the disease**

**Types of patients with IPF**

**8.** According to the ATS/ERS/JRS/ALAT guidelines there appear to be three types of natural histories in patients with IPF (Raghu et al., 2011):

A. Patients with stable disease

B. Patients with slow disease progression

C. Patients with rapid disease progression

In your experience, could you tell us if you agree with this classification of patients with IPF?

| YES |  |
| --- | --- |
| NO |  |

If you agree with this classification, could you please indicate, how would you define these patients in terms of rate of forced vital capacity (FVC) decline per year?

| Type of patient | FVC decline per year (L or % of decline) |
| --- | --- |
| A. Patients with stable disease |  |
| B. Patients with slow disease progression |  |
| C. Patients with rapid disease progression |  |

If you define these patients by other test or parameters, please, describe them detailed below:

| Type of patient | Test / Parameter | Rate / % / … |
| --- | --- | --- |
| A. Patients with stable disease |  |  |
| B. Patients with slow disease progression |  |  |
| C. Patients with rapid disease progression |  |  |

Comments (add here any additional information you might have)

|  |
| --- |

If you disagree with this classification, please indicate how patients with IPF should be classified and how you define them.

|  |
| --- |

Comments (add here any additional information you might have)

|  |
| --- |

**9*.*** In clinical practice, what percentage of the total does each type of patient with IPF represent?

| Type of patient | % of total patients with IPF |
| --- | --- |
| A. Patients with stable disease |  |
| B. Patients with slow disease progression |  |
| C. Patients with rapid disease progression |  |

If you do not agree with this classification, please respond to the question in the next table according to your own classification.

| Type of patient | % of total patients with IPF |
| --- | --- |
|  |  |
|  |  |
|  |  |
|  |  |

Comments (add here any additional information you might have)

|  |
| --- |

**Survival of patients with IPF**

**10.** In your experience, could you tell us how many months a patient with IPF survives on average after diagnosis?

| Type of patient | Survival time (months) |
| --- | --- |
| A. Patients with stable disease |  |
| B. Patients with slow disease progression |  |
| C. Patients with rapid disease progression |  |

If you do not agree with this classification, please respond to the question in the next table according to your own classification.

| Type of patient | Survival time (months) |
| --- | --- |
|  |  |
|  |  |
|  |  |
|  |  |

Comments (add here any additional information you might have)

|  |
| --- |

**Acute exacerbations of IPF**

**11.** Please, indicate below what percentage of patients suffer from acute exacerbation per year (%).

| Type of patient | | Exacerbations | | % | |
| --- | --- | --- | --- | --- | --- |
| Patients with stable disease | | <1 exacerbation per year | |  | |
|  |  | 1 exacerbation per year | |  | |
|  |  | >1 and ≤3 exacerbations per year | |  | |
| Patients with slow disease progression | | <1 exacerbation per year | |  | |
|  |  | 1 exacerbation per year | |  | |
|  |  | >1 and ≤3 exacerbations per year | |  | |
| Patients with rapid disease progression | | <1 exacerbation per year | |  | |
|  |  | 1 exacerbation per year | |  | |
|  |  | >1 and ≤3 exacerbations per year | |  | |

If you do not agree with this classification, please respond to the question in the next table according to your own classification.

| Type of patient | Exacerbations | % |
| --- | --- | --- |
|  | <1 exacerbation per year |  |
|  | 1 exacerbation per year |  |
|  | >1 and ≤3 exacerbations per year |  |
|  | <1 exacerbation per year |  |
|  | 1 exacerbation per year |  |
|  | >1 and ≤3 exacerbations per year |  |
|  | <1 exacerbation per year |  |
|  | 1 exacerbation per year |  |
|  | >1 and ≤3 exacerbations per year |  |

Comments (add here any additional information you might have)

|  |
| --- |

**12.** According to your experience, which proportion of patients with an acute exacerbation die from this acute event?

| Deaths | % |
| --- | --- |
| Patients who die from an acute exacerbation |  |

Of those patients who die from an acute exacerbation, could you indicate which proportion die in the hospital and which proportion die during the next 6 months following the event? (Both percentages must add up to 100%)

| Deaths | % |
| --- | --- |
| Proportion of patients who die from an acute exacerbation in the hospital (as a % of the total number of patients who die from an acute exacerbation) |  |
| Proportion of patients who die from an acute exacerbation during the next 6 months following the event (as a % of the total number of patients who die from an acute exacerbation) |  |

Comments

|  |
| --- |

**13.** How do you differentiate an acute IPF exacerbation from other causes of acute respiratory failure or clinical deterioration? Please, indicate in your experience which additional tests are performed in these patients in order to diagnose an acute exacerbation.

| Test | % of patients with an acute exacerbation |
| --- | --- |
| Computed tomography |  |
| Echocardiography |  |
| Pulmonary scintigraphy |  |
| Lab test |  |
| Complete blood count |  |
| Sedimentation rate |  |
| Hepatic profile |  |
| Creatine phosphokinase (CPK) |  |
| Angiotensin-converting enzyme (ACE) |  |
| Rheumatoid factor |  |
| Antinuclear antibodies |  |
| Microbiology |  |
| Other  ……………. |  |
| Other  ……………. |  |

Comments (add here any additional information you might have)

|  |
| --- |

**14.** According to the ATS/ERS/JRS/ALAT guidelines, acute exacerbations should be treated in most cases with corticosteroids (Raghu et al., 2011). Do you agree with this statement?^[[4]](#footnote-4)^

| YES |  |
| --- | --- |
| NO |  |

If you agree that exacerbations should be treated with corticosteroids, please indicate below which is the current treatment regimen administered to these patients (type of regimen administered (1 active ingredient administered or 2 active ingredients administered sequentially), dose, duration and percentage of patients treated with each option).

| 1 active ingredient administered | Experts who treat patients with each option (%) | Dose administered (mg) | Treatment duration (days) | % of patients treated with each option |
| --- | --- | --- | --- | --- |
| Prednisone |  |  |  |  |
| Methylprednisolone |  |  |  |  |

| 2 active ingredients administered sequentially | Experts who treat patients with each option (%) | Dose administered (mg) | Treatment duration (days) | % of patients treated with each option |
| --- | --- | --- | --- | --- |
| Methylprednisolone |  |  |  |  |
| Prednisone |  |  |  |  |

**15.** If you disagree, please indicate how you treat acute exacerbations in patients with IPF, including both pharmacologic and non-pharmacologic treatments (e.g. anticoagulation, antibiotics, mechanic and non-invasive ventilation).

Could you indicate if you treat acute exacerbations with any of these interventions?^[[5]](#footnote-5)^

| Interventions | Do you treat acute exacerbations with any of these interventions? (YES / NO) |
| --- | --- |
| Antibiotic |  |
| Anticoagulant treatment |  |
| Noninvasive ventilation |  |
| Invasive mechanical ventilation |  |

Comments (add here any additional information you might have)

|  |
| --- |

If you treat acute exacerbations with any of these interventions, please indicate (when necessary) main active ingredient administered, mean dose per day, mean treatment duration and the percentage of patients who are treated with each option.

| Intervention | Active ingredient | Mean dose per day* | Mean treatment duration** | % of patients treated with each intervention |
| --- | --- | --- | --- | --- |
| Antibiotic |  |  |  |  |
| Anticoagulant treatment |  |  |  |  |
| Noninvasive ventilation | - | - |  |  |
| Invasive mechanical ventilation | - | - |  |  |

*Please, indicate units (mg, IU…)

**Please specify the period of time (days, months of treatment ...)

Comments

|  |
| --- |

**16.** In your experience, please indicate healthcare resource consumption during an acute exacerbation and the follow up associated with the exacerbations.

| Resource | % of patients | Mean number (number of visits, days of hospitalization, …) |
| --- | --- | --- |
| Outpatient visits |  |  |
| Pulmonary specialist |  |  |
| Nurse (or other health-care professionals) |  |  |
| Programmed ambulance* |  |  |
| Pulmonary specialist home visit |  |  |
| Nurse home (or other health-care professionals) |  |  |
| Hospitalization |  |  |
| Emergency room visits |  |  |
| Emergency ambulance** |  |  |
| Hospital admissions |  |  |
| Pulmonary department |  |  |
| Intensive care unit |  |  |
| Laboratory Tests |  |  |
| Complete blood count |  |  |
| Sedimentation rate |  |  |
| Hepatic profile |  |  |
| Creatine phosphokinase (CPK) |  |  |
| Angiotensin-converting enzyme (ACE) |  |  |
| Rheumatoid factor |  |  |
| Antinuclear antibodies |  |  |
| Urinalysis |  |  |
| Other tests |  |  |
| Chest X-ray |  |  |
| High resolution computed tomography (HRCT) |  |  |
| Computed tomography pulmonary angiogram |  |  |
| Bronchoscopy^[[6]](#footnote-6)^ |  |  |
| Sputum assessment |  |  |
| Bronchoalveolar lavage |  |  |
| Transbronchial Biopsy |  |  |
| Surgical lung biopsy |  |  |
| Ventilation/perfusion scan |  |  |
| Blood gases |  |  |
| Respiratory function tests: |  |  |
| Spirometry |  |  |
| Bodyplethysmography |  |  |
| Diffusing capacity of carbon monoxide |  |  |
| 6 minute walk test |  |  |
| Other  ……………. |  |  |

*Ambulance programmed in order to transport the patient to the hospital for a programmed visit.

**Emergency ambulance, no programmed ambulance transport.

Comments (add here any additional information you might have)

|  |
| --- |

**Treatment of patients with IPF by stage**

**17.** Based on usual clinical practice, could you tell us what percentages of patients with IPF are administered each of the different types of treatment available? (In the case of anticoagulant, corticosteroid therapies, immunomodulator agents and compassionate drug use / foreign drugs, please specify the active ingredients that are routinely administered to these patients)

| Treatment | Patients with stable disease (%) | Patients with slow disease progression (%) | Patients with rapid disease progression (%) |
| --- | --- | --- | --- |
|  | Or using own categorisation of patients *(please specify in boxes below):* | | |
|  | Category 1:  ……………………. | Category 2:  ……………………. | Category 3:  ……………………. |
| Pharmacological |  |  |  |
| N-Acetylcysteine (both as monotherapy and in combination) |  |  |  |
| Anticoagulants (both as monotherapy and in combination)  Please specify: ……………………….. |  |  |  |
| Systemic corticosteroids (both as monotherapy and in combination)  Please specify: ……………………….. |  |  |  |
| Immunomudulator agents, e.g. azathioprine (both as monotherapy and in combination)  Please specify: ……………………….. |  |  |  |
| Long-term oxygen therapy |  |  |  |
| Non-pharmacological |  |  |  |
| Lung Transplantation |  |  |  |
| Single-lung transplant |  |  |  |
| Double-lung transplant |  |  |  |
| Pulmonary Rehabilitation |  |  |  |
| Compassionate drug use* /Foreign drug** (ex. pirfenidone)  ……………………….. |  |  |  |
| Treatment within a clinical trial  ……………………….. |  |  |  |
| Other  ……………………….. |  |  |  |
| Other  ……………………….. |  |  |  |

*Drugs non-commercialized in Spain which are in clinical research (usually for diseases that have no effective treatment available) or commercialized drugs that are used for indications non approved and included in the Summary of product characteristics

**Drugs non-commercialized that a Spanish physician wants to prescribe and makes a request to the Spanish Agency of Drugs, normally as compassionate use'.

Comments (add here any additional information you might have)

|  |
| --- |

**Pharmacotherapy**

**18.** In your experience, if treated with one of these different drug treatments available, for how long (per year) and at which dose do you treat each type of patient? (In the case of anticoagulant, corticosteroid therapies, immunomodulator agents and compassionate drug use / Foreign drugs, please specify the active ingredients that are routinely administered to these patients).

| Pharmacotherapy | Patients with stable disease | | | Patients with slow disease progression | | | Patients with rapid disease progression | | |
| --- | --- | --- | --- | --- | --- | --- | --- | --- | --- |
|  | Or using own categorisation of patients *(please specify in boxes below):* | | | | | | | | |
|  | Category 1:  ………………. | | | Category 2:  ………………. | | | Category 3:  ………………. | | |
|  | Active ingredient | Mean dose per day* | Mean duration per year** | Active ingredient | Mean dose per day * | Mean duration per year** | Active ingredient | Mean dose per day * | Mean duration per year** |
| N-Acetylcysteine |  |  |  |  |  |  |  |  |  |
| Anticoagulants |  |  |  |  |  |  |  |  |  |
| Systemic corticosteroids |  |  |  |  |  |  |  |  |  |
| Immunomudulator agents |  |  |  |  |  |  |  |  |  |
| Compassionate drug use / Foreign drug (ex. pirfenidone) |  |  |  |  |  |  |  |  |  |
| Long-term oxygen therapy |  |  |  |  |  |  |  |  |  |
| Other  ……………….. |  |  |  |  |  |  |  |  |  |
| Other  ……………….. |  |  |  |  |  |  |  |  |  |
| Other  ……………….. |  |  |  |  |  |  |  |  |  |

*Please, indicate units (mg, IU…)

**Please specify the period of time (days, months of treatment ...)

Comments (add here any additional information you might have)

|  |
| --- |

**Non-pharmacological therapies**

**19.** In your experience, could you detail the resource use of non-pharmacological therapies (home medical visits, nursing home, ...)?

| Non-pharmacological therapy | Resource use (number of rehabilitations sessions, home medical visits, nursing home, ...) |
| --- | --- |
| Pulmonary Rehabilitation |  |
| Other  ................... |  |
| Other  ................... |  |

Comments (add here any additional information you might have)

|  |
| --- |

**Adverse events of pharmacological treatments^[[7]](#footnote-7)^**

**20.** With respect to the potential adverse events of available drug treatments right below we have listed those reported more frequently. In your experience, for each treatment, please, could you indicate how many IPF patients (%) suffer from each adverse event with each treatment and severity grade?. You can also add those adverse events not included in the list.

**N-Acetylcysteine**

| Adverse events (AE) | % of patients | | |
| --- | --- | --- | --- |
|  | Grade I-II | Grade III | Grade IV |
| Epigastric pain |  |  |  |
| Digestive intolerance |  |  |  |
| Dyspepsia |  |  |  |
| Reflux |  |  |  |
| Nausea |  |  |  |
|  |  |  |  |
|  |  |  |  |
|  |  |  |  |

**Anticoagulants**

| Adverse events (AE) | % of patients | | |
| --- | --- | --- | --- |
|  | Grade I-II | Grade III | Grade IV |
| Haemorrhage |  |  |  |
| Haematoma |  |  |  |
|  |  |  |  |
|  |  |  |  |
|  |  |  |  |

**Systemic corticosteroids**

| Adverse events (AE) | % of patients | | |
| --- | --- | --- | --- |
|  | Grade I-II | Grade III | Grade IV |
| Osteoporosis |  |  |  |
| Opportunistic infections |  |  |  |
| Edema |  |  |  |
| Myopathy |  |  |  |
| Hyperglycemia |  |  |  |
| Cushing syndrome |  |  |  |
| Compression fractures |  |  |  |
| Diabetes |  |  |  |
| Arterial hypertension |  |  |  |
| Cataract |  |  |  |
| Digestive intolerance |  |  |  |
|  |  |  |  |
|  |  |  |  |
|  |  |  |  |

**Immunomodulator agents**

| Adverse events (AE) | Grade I-II | Grade III | Grade IV |
| --- | --- | --- | --- |
| Opportunistic infections |  |  |  |
| Hepatotoxicity |  |  |  |
| Myelotoxicity |  |  |  |
| Leukopenia |  |  |  |
| Anemia |  |  |  |
|  |  |  |  |
|  |  |  |  |
|  |  |  |  |

**Pirfenidone (Compassionate drug use / Foreign drug)**

| Adverse events (AE) | Grade I-II | Grade III | Grade IV |
| --- | --- | --- | --- |
| Photosensitivity |  |  |  |
| Epigastric pain |  |  |  |
| Dizziness |  |  |  |
| Skin reactions |  |  |  |
| Anorexia |  |  |  |
| Nauseas |  |  |  |
| Asthenia |  |  |  |
| Digestive intolerance |  |  |  |
|  |  |  |  |
|  |  |  |  |
|  |  |  |  |

**Long-term oxygen therapy**

| Adverse events (AE) | Grade I-II | Grade III | Grade IV |
| --- | --- | --- | --- |
| Nasal dryness |  |  |  |
| Epistaxis |  |  |  |
| Dry mouth |  |  |  |
| Hypercapnia |  |  |  |
|  |  |  |  |
|  |  |  |  |
|  |  |  |  |

Comments

|  |
| --- |

**Adverse events of non-pharmacological treatments**

**21.** With respect to the potential adverse events of non-pharmacological therapies, in your experience, for each non pharmacological therapy, please state the adverse event associated with the treatment that has the greatest economic impact

| Non-pharmacological therapy | Adverse events (AE) | % of patients treated with each drug who develop the AE |
| --- | --- | --- |
| Pulmonary Rehabilitation |  |  |
| Other  ................... |  |  |
| Other  ................... |  |  |

Comments (add here any additional information you might have)

|  |
| --- |

**Monitoring by stage**

**22.** According to usual clinical practice, please indicate the frequency of use of each resource involved in the clinical control and follow-up of patients with IPF over a 3-month period (number of visits by patients to the pulmonary medicine department, number of tests, x-rays, etc.).

| Resource | Patients with stable disease | Patients with slow disease progression | Patients with rapid disease progression |
| --- | --- | --- | --- |
|  | Or using own categorisation of patients *(please specify in boxes below):* | | |
|  | Category 1:  ………………. | Category 2:  ………………. | Category 3:  ………………. |
| Outpatient visits |  |  |  |
| Pulmonary specialist |  |  |  |
| Nurse (or other health-care professionals) |  |  |  |
| Pulmonary specialist home visit |  |  |  |
| Nurse home (or other health-care professionals) |  |  |  |
| Hospitalization |  |  |  |
| Emergency room visits |  |  |  |
| Hospital admissions |  |  |  |
| Pulmonary department (indicate number of hospitalizations and duration (days)) |  |  |  |
| Intensive care unit (indicate number of hospitalizations and duration (days)) |  |  |  |
| Laboratory Tests |  |  |  |
| Complete blood count |  |  |  |
| Sedimentation rate |  |  |  |
| Hepatic profile |  |  |  |
| Creatine phosphokinase (CPK) |  |  |  |
| Angiotensin-converting enzyme (ACE) |  |  |  |
| Rheumatoid factor |  |  |  |
| Antinuclear antibodies |  |  |  |
| Urinalysis |  |  |  |
| Other tests |  |  |  |
| Chest X-ray |  |  |  |
| High resolution computed tomography (HRCT) |  |  |  |
| Computed tomography pulmonary angiogram |  |  |  |
| Bronchoscopy^[[8]](#footnote-8)^ |  |  |  |
| Sputum assessment |  |  |  |
| Bronchoalveolar lavage |  |  |  |
| Transbronchial Biopsy |  |  |  |
| Surgical lung biopsy |  |  |  |
| Ventilation/perfusion scan |  |  |  |
| Blood gases |  |  |  |
| Respiratory function tests: |  |  |  |
| Spirometry |  |  |  |
| Bodyplethysmography |  |  |  |
| Diffusing capacity of carbon monoxide |  |  |  |
| 6 minute walk test |  |  |  |
| Other  ……………. |  |  |  |
| Other  ……………. |  |  |  |

Comments (add here any additional information you might have)

|  |
| --- |

**Comorbidities**

**23.** Below are some comorbidities that may occur in patients with IPF (Collard et al., 2012). Please indicate what percentages of patients have each comorbidity. If you think there are other comorbidities that should be included in the study, please list them at the end of the table.

| Comorbidity | Patients with stable disease | Patients with slow disease progression | Patients with rapid disease progression |
| --- | --- | --- | --- |
|  | Or using own categorisation of patients *(please specify in boxes below):* | | |
|  | Category 1:  ………………. | Category 2:  ………………. | Category 3:  ………………. |
| Pulmonary infection |  |  |  |
| Coronary artery disease (exclusive of MI) |  |  |  |
| Diabetes |  |  |  |
| Heart failure |  |  |  |
| Chronic bronchitis |  |  |  |
| Atrial fibrillation |  |  |  |
| Fatigue |  |  |  |
| Cerebrovascular disease |  |  |  |
| Asthma |  |  |  |
| Emphysema |  |  |  |
| Gastroesophageal reflux disease |  |  |  |
| Obstructive sleep apnoea |  |  |  |
| Depression |  |  |  |
| Myocardial infarction |  |  |  |
| Lung cancer |  |  |  |
| Pulmonary embolism |  |  |  |
| Pulmonary hypertension |  |  |  |
| Obesity |  |  |  |
| Osteoporosis |  |  |  |
| Cataracts |  |  |  |
| Deep vein thrombosis |  |  |  |
| Other  ……………………… |  |  |  |
| Other  ……………………… |  |  |  |

Comments (add here any additional information you might have)

|  |
| --- |

**End-of-life care**

**24.** In your experience, at what point is end-of-life/palliative care initiated in IPF patients and for how long palliative care is administered? (e.g. mean months of end-of-life/palliative care).

|  |
| --- |

Comments (add here any additional information you might have)

|  |
| --- |

Right below, palliative treatments have been listed. In your experience, please indicate, main active ingredient administered (when necessary), mean dose per day, mean treatment duration and the percentage of patients who are treated with each option.

| Type of end-of-life/palliative care received by IPF patient | Active ingredient | % of patients treated with each intervention | Mean dose per day* | Mean treatment duration** |
| --- | --- | --- | --- | --- |
| Peripheral analgesia | - | - | - | - |
| Paracetamol | - |  |  |  |
| Nonsteroidal anti-inflammatory drugs (NSAIDs) | - |  |  |  |
| Other |  |  |  |  |
| Other |  |  |  |  |
| Weak opioid | - | - | - | - |
| Codeine | - |  |  |  |
| Dihydrocodeine | - |  |  |  |
| Other |  |  |  |  |
| Other |  |  |  |  |
| Strong opioids | - | - | - | - |
| Morphine | - |  |  |  |
| Buprenorphine patches | - |  |  |  |
| Fentanyl transdermal patch | - |  |  |  |
| Other |  |  |  |  |
| Other |  |  |  |  |
| Co-analgesics | - | - | - | - |
| Corticosteroids |  |  |  |  |
| Antidepressant |  |  |  |  |
| Anticonvulsant |  |  |  |  |
| Phenothiazines |  |  |  |  |
| Other treatments |  |  |  |  |
|  |  |  |  |  |
|  |  |  |  |  |
|  |  |  |  |  |

Comments

|  |
| --- |

**25.** Regarding resource consumption associated with palliative care, please could you indicate mean number of visits undertaken in order to administer and control palliative care at the end-of-life care?

| Outpatient visits | Mean number of visits during palliative care |
| --- | --- |
| Pulmonary specialist |  |
| Nurse (or other health-care professionals) |  |
| Pulmonary specialist home visit |  |
| Nurse home (or other health-care professionals) |  |
| Pulmonary specialist home visit |  |
| Other  …………………………… |  |

Comments

|  |
| --- |

**Impact on patients, families and/or caregivers**

**Impact on patients**

**26.** According to your experience, could you

How many retired patients are when they develop the disease?

| % of retired patients with stable disease | % of retired patients with slow disease progression | % of retired patients with rapid disease progression |
| --- | --- | --- |
| Or using own categorisation of patients *(please specify in boxes below):* | | |
| Category 1:  ………………. | Category 2:  ………………. | Category 3:  ………………. |
|  |  |  |

**27.** Of those patients who keep employed during their illness, how many sick leaves they suffered per year and how many days it lasts (average) (sick leaves include both due to acute exacerbations and those that do not).

|  | Patients with stable disease | Patients with slow disease progression | Patients with rapid disease progression |
| --- | --- | --- | --- |
|  | Or using own categorisation of patients *(please specify in boxes below):* | | |
|  | Category 1:  ………………. | Category 2:  ………………. | Category 3:  ………………. |
| Sick leaves per year |  |  |  |
| Duration of each sick leave (days) |  |  |  |

Comments

|  |
| --- |

**28.** Percentage of IPF patients who are total permanent disability

| % of total permanent disability patients with slow disease progression | % of total permanent disability patients with stable disease | % of total permanent disability patients with rapid disease progression |
| --- | --- | --- |
|  |  |  |

Comments

|  |
| --- |

**Impact on relatives and caregivers**

**29.** Percentage of working relatives of patients with IPF who lose working days in order to take care of them.

| % of working relatives who lose working days in order to care for patients with IPF | | |
| --- | --- | --- |
| Patients with stable disease | Patients with slow disease progression | Patients with rapid disease progression |
| Or using own categorisation of patients *(please specify in boxes below):* | | |
| Category 1:  ………………. | Category 2:  ………………. | Category 3:  ………………. |
|  |  |  |

Comments

|  |
| --- |

**30.** Of relatives who lose working days to care for a patient with IPF, percentage that accompany the patient on follow-up medical visits for the disease

| % of working relatives who accompany patients with IPF to medical follow-up visits for the disease | | |
| --- | --- | --- |
| Patients with stable | Patients with slow disease progression disease | Patients with rapid disease progression |
| Or using own categorisation of patients *(please specify in boxes below):* | | |
| Category 1:  ………………. | Category 2:  ………………. | Category 3:  ………………. |
|  |  |  |

Comments

|  |
| --- |

**31.** Percentage of working relatives who lose working days to take care of IPF patients with temporarily disability (hospitalization, hospital-at-home…)

| % of working relatives who lose working days to care for IPF patients with temporary disability | | |
| --- | --- | --- |
| Patients with stable | Patients with slow disease progression disease | Patients with rapid disease progression |
| Or using own categorisation of patients *(please specify in boxes below):* | | |
| Category 1:  ………………. | Category 2:  ………………. | Category 3:  ………………. |
|  |  |  |

Comments

|  |
| --- |

**32.** Percentage of working relatives who lose working days to care for IPF patients with permanent disability.

| % of working relatives who lose working days to care for IPF patients with permanent disability | | |
| --- | --- | --- |
| Patients with stable | Patients with slow disease progression disease | Patients with rapid disease progression |
| Or using own categorisation of patients *(please specify in boxes below):* | | |
| Category 1:  ………………. | Category 2:  ………………. | Category 3:  ………………. |
|  |  |  |

Comments

|  |
| --- |

**33.** Percentage of patients with IPF who receive care from paid caregivers and how long this care lasts.

| % of patients with IPF who receive care from paid caregivers | | |
| --- | --- | --- |
| Patients with stable disease | Patients with slow disease progression | Patients with rapid disease progression |
| Or using own categorisation of patients *(please specify in boxes below):* | | |
| Category 1:  ………………. | Category 2:  ………………. | Category 3:  ………………. |
|  |  |  |
| Duration of care received from paid caregivers | | |
|  |  |  |

Comments

|  |
| --- |

**Additional comments**

In the space below, please add any comments you consider relevant and which have not been included in this questionnaire. Especially let us know if you think there is any aspect of the study that should be discussed in detail.

**THANK YOU FOR YOUR COOPERATION**

**References**

- Ancochea J, Antón E, Casanova A. (New therapeutic strategies in idiopathic pulmonary fibrosis). Arch Bronconeumol. 2004;40 Suppl 6:16-22.
- Collard HR, Ward AJ, Lanes S, Cortney Hayflinger D, Rosenberg DM, Hunsche E. Burden of illness in idiopathic pulmonary fibrosis. J Med Econ. 2012;15(5):829-35.
- Consenso para el diagnóstico de las neumonías intersticiales idiopáticas. Arch Bronconeumol.2010; 46(Suppl.5) :2-21
- Marcos PJ, Valenzuela C, Ancochea J. De la exclusión a la incertidumbre. El recorrido hacia el diagnóstico de la fibrosis pulmonar idiopática. Arch Bronconeumol. 2012;48(Supl 2):7-12.
- Mora G, Romero A. Guía para pacientes con fibrosis pulmonar idiopática 2012. Diponible en: http://www.separ.es/biblioteca-1/Biblioteca-para-Profesionales/consensos
- Raghu G, Collard HR, Egan JJ, Martinez FJ, Behr J, Brown KK et al. An official ATS/ERS/JRS/ALAT statement: idiopathic pulmonary fibrosis: evidence-based guidelines for diagnosis and management. Am J Respir Crit Care Med. 2011;183(6):788-824.
- Vaquero M. Neumonías intersticiales idiopáticas pediátricas y del adulto: conceptos actuales sobre su clasificación y patogénesis. Idiopathic interstitial pneumonia in children and adults: actual criteria on classification and pathogenesis. Rev Esp Patol. 2008;41:85-98.
- Xaubet A, Ancochea J, Blanquer R, Montero C, Morell F, Rodríguez Becerra E et al. Diagnóstico y tratamiento de las enfermedades pulmonares intersticiales difusas. Diagnosis and treatment of diffuse interstitial lung diseases. Arch Bronconeumol. 2003;39:580-600.
- Xaubet A, Ancochea J. Actualización en fibrosis pulmonar idiopática: Introducción. Arch Bronconeumol. 2012;48(Suppl 2):1.
- Xaubet A. Consideraciones sobre la nueva clasificación de las neumopatías intersticiales difusas. Clinical comments on the new classification of diffuse interstitial lung diseases. Med Clin (Barc). 2003;121:389-95.
- King TE Jr, Pardo A, Selman M. Idiopathic pulmonary fibrosis. Lancet. 2011;378(9807):1949-61.
- Ley B, Collard HR, King TE Jr. Clinical course and prediction of survival in idiopathic pulmonary fibrosis. Am J Respir Crit Care Med. 2011;183(4):431-40.
- Morell F, Reyes L, Doménech G, De Gracia J, Majó J, Ferrer J. (Diagnoses and diagnostic procedures in 500 consecutive patients with clinical suspicion of interstitial lung disease). Arch Bronconeumol. 2008;44(4):185-91.

1. To calculate prevalence: Number of patients visited per year / Hospital reference population area [↑](#footnote-ref-1)
2. To calculate incidence: Number of new patients visited per year / Hospital reference population area [↑](#footnote-ref-2)
3. Bronchoscopy, bronchoalveolar lavage and transbronchial biopsy are usually carried out at the same time [↑](#footnote-ref-3)
4. In the first questionnaire, experts were asked whether they agreed with this statement. All experts agreed that all patients with acute exacerbations should be treated with corticosteroids. This question regarding corticosteroid treatment was asked through the second questionnaire in order to describe the therapeutic algorithm followed by the experts in Spain. Due to the inconsistencies found, it was decided to ask this question by phone call. [↑](#footnote-ref-4)
5. In addition to corticosteroids, 27% of the experts stated in the first questionnaire that they treated patients with acute exacerbations with other drugs. Therefore, in the second questionnaire the experts were asked deeper if they treated patients with any of the treatments referred to in the first questionnaire and, if they did, what percentage of patients received them, at what doses and for how long on average [↑](#footnote-ref-5)
6. Bronchoscopy, bronchoalveolar lavage and transbronchial biopsy are usually carried out at the same time [↑](#footnote-ref-6)
7. For each AE, if possible, it would be advisable to ask about the treatment of Grade I-II and Grade III AE. [↑](#footnote-ref-7)
8. Bronchoscopy, bronchoalveolar lavage and transbronchial biopsy are usually carried out at the same time [↑](#footnote-ref-8)
