# Supplementary material for: Treatment patterns, resource use and costs of idiopathic pulmonary fibrosis in Spain – results of a Delphi Panel
Source: BMC Pulm Med. 2016 Jan 12;16:7. doi: 10.1186/s12890-016-0168-6 (PMC4710031; doi:10.1186/s12890-016-0168-6)
Supplement: Additional file 2: Table S1. — Unit costs used in the study. (DOCX 23 kb) [file 12890_2016_168_MOESM2_ESM.docx]

Additional file 2 to accompany research article “Treatment patterns, resource use and costs of idiopathic pulmonary fibrosis in Spain – results of a Delphi Panel” by Ferran Morell et al

Table S1 Unit costs used in the study

|  | **Unit costs  (€ 2013)** | **Source** |
| --- | --- | --- |
| **Visits** |  |  |
| Primary care | €29.05 | ^10^ |
| Pulmonary medicine department | €74.66 | ^10^ |
| Pulmonary specialist home visit | €74.66 | ^10^ |
| Nurse (or other healthcare professional) | €17.32 | ^10^ |
| Home nurse (or other healthcare professional) | €34.99 | ^10^ |
| General practitioner home visits | € 5.68 | ^10^ |
| Elective ambulance | €16.90 | ^10^ |
| **Hospitalisation** |  |  |
| Emergency room visits | €178.19 | ^10^ |
| Emergency ambulance | €317.96 | ^10^ |
| Hospital admissions - Pulmonary department | €516.14 | ^10^ |
| Hospital admissions - Intensive care unit | €1,687.37 | ^10^ |
| **Laboratory tests** |  |  |
| Complete blood count | €4.16 | ^10^ |
| Sedimentation rate | €1.98 | ^10^ |
| Hepatic profile | €25.88 | ^10^ |
| Creatine phosphokinase | €4.61 | ^10^ |
| Angiotensin-converting enzyme | €19.40 | ^10^ |

| Rheumatoid factor | €9.20 | ^10^ |
| --- | --- | --- |
| Antinuclear antibodies | €28.26 | ^10^ |
| Urinalysis | €6.02 | ^10^ |
| Microbiology | €19.30 | ^10^ |
| Procalcitonin | €35.19 | ^10^ |
| **Respiratory function tests** |  |  |
| Pulmonary function test (lung function test and spirometry) | €163.64 | ^10^ |
| Spirometry | €34.66 | ^10^ |
| Body plethysmography | €45.35 | ^10^ |
| Diffusing capacity of carbon monoxide | €83.63 | ^10^ |
| 6-minute walk test | €143.70 | ^10^ |
| Specific bronchial provocation test | €99.91 | ^10^ |
| Ergometry | €116.71 | ^10^ |
| **Other tests** | | |
| Chest X-ray | €17.30 | ^10^ |
| Bronchoscopy | €177.88 | ^10^ |
| Bronchoscopy + bronchoalveolar lavage | €233.70 | ^10^ |
| Bronchoscopy + bronchoalveolar lavage + transbronchial biopsy | €280.28 | ^10^ |
| Bronchoscopy + transbronchial biopsy | €224.46 | ^10^ |
| Surgical lung biopsy | €7,014.72 | ^10^ |
| Sputum assessment | €69.57 | ^10^ |
| Pulmonary ventilation/perfusion scan | €294.21 | ^10^ |
| Blood gases | €8.23 | ^10^ |
| Computed tomography | €153.31 | ^10^ |
| High resolution computed tomography | €212.06 | ^10^ |
| Computed tomography pulmonary angiogram | €150.42 | ^10^ |
| Echocardiography | €81.25 | ^10^ |
| **Pharmacological treatment (active ingredient) (cost per unit)** | | |
| Azathioprine | €0.01 / mg | ^9^ |
| N-acetylcysteine | €0.0006 / mg | ^9^ |
| Pirfenidone | €0.04 / mg | ^a^ |
| Omeprazole / Pantoprazole | €0.01 / mg | ^9^ |
| ***Corticosteroid*** |  |  |
| Methylprednisolone | €0.07 / mg | ^9^ |
| Prednisone | €0.01 / mg | ^9^ |
| ***Antibiotic*** |  |  |
| Amoxicillin/clavulanic acid | €0.0003 / mg | ^9^ |
| Levofloxacin | €0.003 / mg | ^9^ |
| Voriconazole | €0.17 / mg | ^9^ |
| Piperacillin / Tazobactam | €0.002 / mg | ^9^ |
| Moxifloxacin | €0.02 / mg | ^9^ |
| Imipenem/cilastatin | €0.013 / mg | ^9^ |
| Ceftriaxone | €0.004 / mg | ^9^ |
| ***Anticoagulant*** |  |  |
| Tinzaparin sodium | €0.0003 / UI | ^9^ |
| Bemiparin sodium | €0.001 / UI | ^9^ |
| Enoxaparin | €0.001 / UI | ^9^ |
| Coumarin anticoagulants | €0.02 / mg | ^9^ |
| Nadroparin | €0.001 / UI | ^9^ |
| ***Peripheral analgesia*** |  |  |
| Paracetamol | €0.01 / mg | ^9^ |
| Nonsteroidal anti-inflammatory drugs | €0.0003 / mg | ^9^ |
| ***Weak opioid*** |  |  |
| Codeine | €0.01 / mg | ^9^ |
| Dihydrocodeine | €0.01 / mg | ^9^ |
| ***Strong opioid*** |  |  |
| Morphine | €0.02 / mg | ^9^ |
| Buprenorphine patch | €0.66 / mg | ^9^ |
| Fentanyl transdermal patch | €0.47 / mg | ^9^ |
| ***Co-analgesic*** |  |  |
| Antidepressant - paroxetine | €0.01 / mg | ^9^ |
| **Noninvasive ventilation (per day)** | €9.48 | ^10^ |
| **Invasive mechanical ventilation** | - | ^b^ |
| Long-term oxygen therapy (per day) | €4.04 | ^9^ |
| Lung transplantation (single) | €109,129.01 | ^10^ |
| Pulmonary rehabilitation (session) | €25.24 | ^10^ |

^a^As pirfenidone was not reimbursed in Spain at the time the study was conducted, the cost was assumed to be the same as that referred to in a document developed by a Spanish hospital for its use as a foreign drug. The cost stated in this report is the cost approved in Germany and Austria.

^b^Invasive ventilation is carried out during hospitalisation. The cost was not included to avoid double counting.
